# Mass mortality events of autochthonous faunas in a Lower Cretaceous Gondwanan Lagerstätte

**DOI:** 10.1038/s41598-021-85953-5

**Published:** 2021-03-26

**Authors:** Arianny P. Storari, Taissa Rodrigues, Renan A. M. Bantim, Flaviana J. Lima, Antonio A. F. Saraiva

**Affiliations:** 1 Laboratório de Paleontologia, Departamento de Ciências Biológicas, Centro de Ciências Humanas e Naturais, Universidade Federal do Espírito Santo, Vitória, Espírito Santo Brazil; 2 Laboratório de Paleontologia da URCA-LPU, Centro de Ciências Biológicas e da Saúde, Universidade Regional do Cariri, Crato, Ceará Brazil

**Keywords:** Entomology, Palaeoclimate, Palaeoecology

## Abstract

Mass mortality events are unusual in the Crato Formation. Although mayflies’ accumulations have been previously reported from that unit, they lacked crucial stratigraphic data. Here we provide the first taphonomic analysis of a mayfly mass mortality event, from a layer 285 cm from the top of the Formation, with 40 larvae, and an overview of the general biological community structure of a three meters deep excavated profile. The only other autochthonous taxon observed in the mayfly mortality layer was the gonorynchiform fish *Dastilbe*. The larvae and fishes were smaller than usual in the layer 285 cm, suggesting that they lived in a shallow water column. Their excellent preservation and a lack of preferential orientation in the samples suggest an absence of significant transport. All mayflies belong to the Hexagenitidae, whose larvae lived in quiet waters. We also recovered allochthonous taxa in that layer indicative of drier weather conditions. Adjacent layers presented crystals and pseudomorphs of halite, suggesting drought and high salinity. In other layers, *Dastilbe* juveniles were often found in mass mortality events, associated with a richer biota. Our findings support the hypothesis that the Crato Formation’s palaeolake probably experienced seasonal high evaporation, caused by the hot climate tending to aridity, affecting the few autochthonous fauna that managed to live in this setting.

## Introduction

The Crato Formation (northeastern Brazil) is a lithostratigraphic unit well-known by its fossiliferous laminated limestones. It represents a paleoenvironment composed of a lacustrine complex approximately 100 km × 50 km in total area ^1,2^, with freshwater constituting the superficial and marginal portions of the lakes^1–3^. The unit was deposited during the Upper Aptian, Lower Cretaceous^4^, under a stratified water column, with relatively well oxygenated upper layers, and reportedly anoxic lower layers^2,5,6^.

Insects preserved in carbonates often belong to groups that rely on water for habitat, hunting, or laying eggs^7^. Possibly, the aquatic insect fossils (e.g. Ephemeroptera) of this deposit represent both autochthonous and allochthonous taxa, as some may have been transported from the lotic to the lentic regions^8^. Taxa that not necessarily depended on lotic environments, such as the autochthonous Hexagenitidae larvae (Ephemeroptera) and *Dastilbe* fish^9–11^, stands out as dominant groups in the Crato Formation^8,10,12^.

Mass mortality events are unusual in the Crato Formation, but assemblages of mayflies’ larvae found in its yellowish limestones have been previously reported as representing such episodes^9,13^. Small accumulations of more than three insects nearby on the same bedding plane are known for this unit, but such aggregations are rare^14^. Although Menon and Martill^8^ alleged there was no clear evidence for mass mortality events in the Crato Formation, Martins-Neto and Gallego^15^ stated that many of the taxa that depended on freshwater to live and/or reproduce probably suffered mass mortality events, that could have been caused by a periodic increase of H_2_S^16^. According to Martins-Neto^13^, mayflies’ mortality horizons during the Cretaceous are observed in a geographic range covering Mongolia, north of China, Transbaikalia (Russia), northwest Africa, and the northeast of Brazil, having as a possible cause the tropical climate tending to aridity. However, previous observations on mayfly mass mortality events within the Crato Formation lacked crucial stratigraphic control. Here we provide the first taphonomic analysis of specimens collected from controlled excavations, the first of their kind for that unit.

## Material and methods

### Geological setting

The Araripe Basin is located in Brazil’s northeastern region and presents outcrops in three different states: southwestern Ceará, northwestern Pernambuco, and eastern Piauí^17^. Among the Cretaceous deposits, the Santana Group is a depositional sequence associated with the South Atlantic opening. It comprises, from bottom to top, the Barbalha, Crato, Ipubi, and Romualdo Formations^18^. From these, the Crato Formation represents a stratigraphic sequence of lacustrine deposits with a predominance of carbonates. It is constituted by six units named, from bottom to top, C1–C6, interleaved by sandstones, siltstones, and shales^19^. These six carbonate packages can be found from the municipality of Santana do Cariri until near Porteiras, both in Ceará, in a series of laminated limestone outcrops on the Araripe Plateau, where they are commonly located in commercial quarries or river margins^20^. More detailed geological and sedimentological information about the Crato Formation can be found in Assine^18^, and Viana and Neumann^20^.

### Excavation and collection

**Figure 1:**
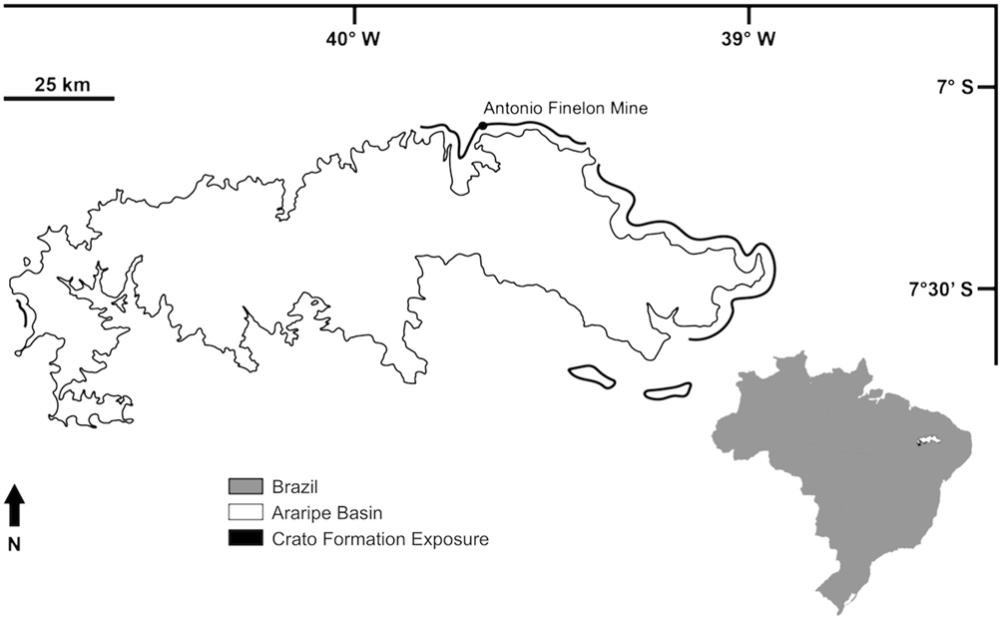
Locality map. Antônio Finelon Mine, Nova Olinda municipality, Ceará State, Brazil. Outcrops of the Crato Formation and of the Araripe Basin are also indicated.

**Figure 2:**
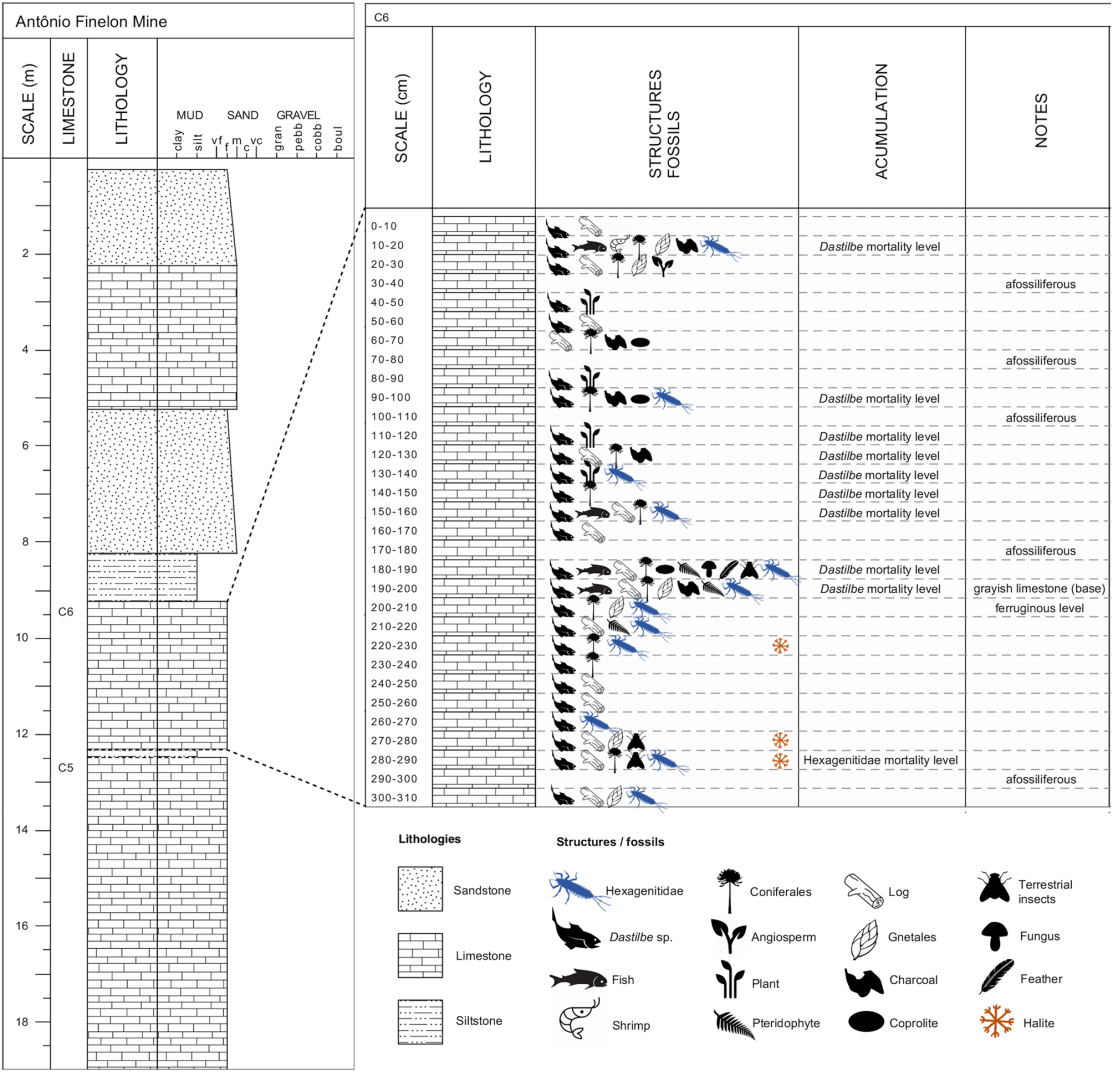
Excavation profile of an outcrop of the Crato Formation. Antônio Finelon Mine, Nova Olinda municipality, Ceará State, Brazil. On the right, the section excavated of 3.10 m in depth, at level C6, evidencing the lithostratigraphic position of the fossil assemblage and levels with fossil accumulation.

**Figure 3:**
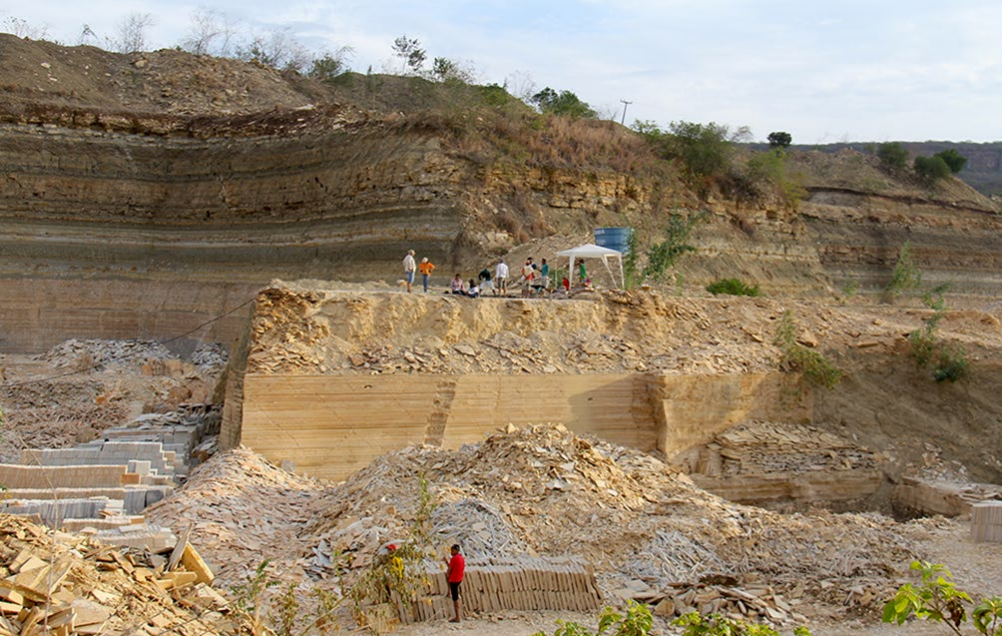
Photograph of the controlled excavations at Antônio Finelon Mine. Nova Olinda municipality, Ceará State, Brazil.

Controlled excavations were conducted by the group of palaeontologists of the Universidade Regional do Cariri (URCA) in an outcrop of the Crato Formation at the Antônio Finelon Mine (S 07° 07′ 22.5″ and W 39° 42′ 01″) in Nova Olinda municipality, Ceará State, Brazil (Fig. 1). The quarry’s surface was divided into 5.0 m^2^ × 2.0 m^2^ quadrants and was excavated until the base of the Formation, in total, three meters in depth (Figs. 2 and 3). A sequence number was attributed on a field form for all collected fossils, and the following information was assessed: the place of the collection (distance from the top of the excavation); type of fossil (to the least inclusive taxonomic group possible); integrity (complete, incomplete or fragment); preservation type (compressed, impression, or 3D); fossil length (cm); fossil width (cm); fossil orientation (azimuth) and other observations of interest (such as sedimentological). The collected fossils were deposited in the Paleontological Collection of URCA (LPU) in Crato municipality, and the Museu de Paleontologia Plácido Cidade Nuvens (MPPCN) in Santana do Cariri municipality, both in Ceará State, Brazil.

## Results

### Mayflies' mass mortality layer

**Figure 4:**
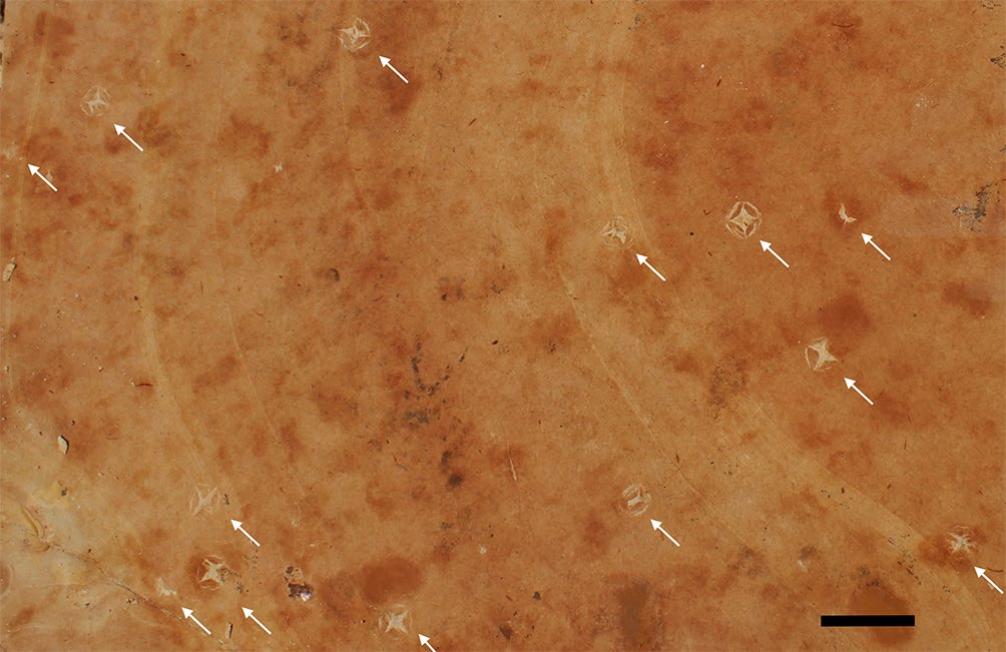
Halite crystals. Halite crystals recovered from layer 288 cm. White arrows point to crystals. Scale bar 20 mm.

A layer collected at 285 cm from the top of the Formation, belonging to top-level carbonate C6 sensu Neumann and Cabrera^19^ and composed of yellowish limestone, presented evidence of at least one mass mortality event, with 40 mayflies’ larvae recovered over 5.0 × 2.0 m^2^. Its over and underlying layers, at 274.5 and 288 cm, respectively, presented halite crystals and pseudomorphs (Fig. 4). The only other autochthonous taxon observed at the 285 cm level was 18 specimens of the gonorynchiform fish *Dastilbe*.

All mayflies’ larvae from that level belong to the extinct family Hexagenitidae and could be identified as a monospecific assemblage of *Protoligoneuria limai* Demoulin, 1955 due to the diagnostic enlarged seventh gill^21^. Martins-Neto^9^ classified *Protoligoneuria* larvae into ontogenetic categories: specimens with a body length between 0.1 and 1 cm as young and larvae up to 1.2–1.6 cm as mature. The body length of the specimens recovered from this level is consistent with the former (Supplementary Table S1). At the 285 cm layer, the larvae are mostly young, evidenced by the wing pad’s absence; therefore, this accumulation represents a selective death^22^. When the adults emerge *en-masse*, a mass mortality event can occur^23^, but it was probably not this case, since most larvae were not mature enough to moult into adulthood.

**Figure 5:**
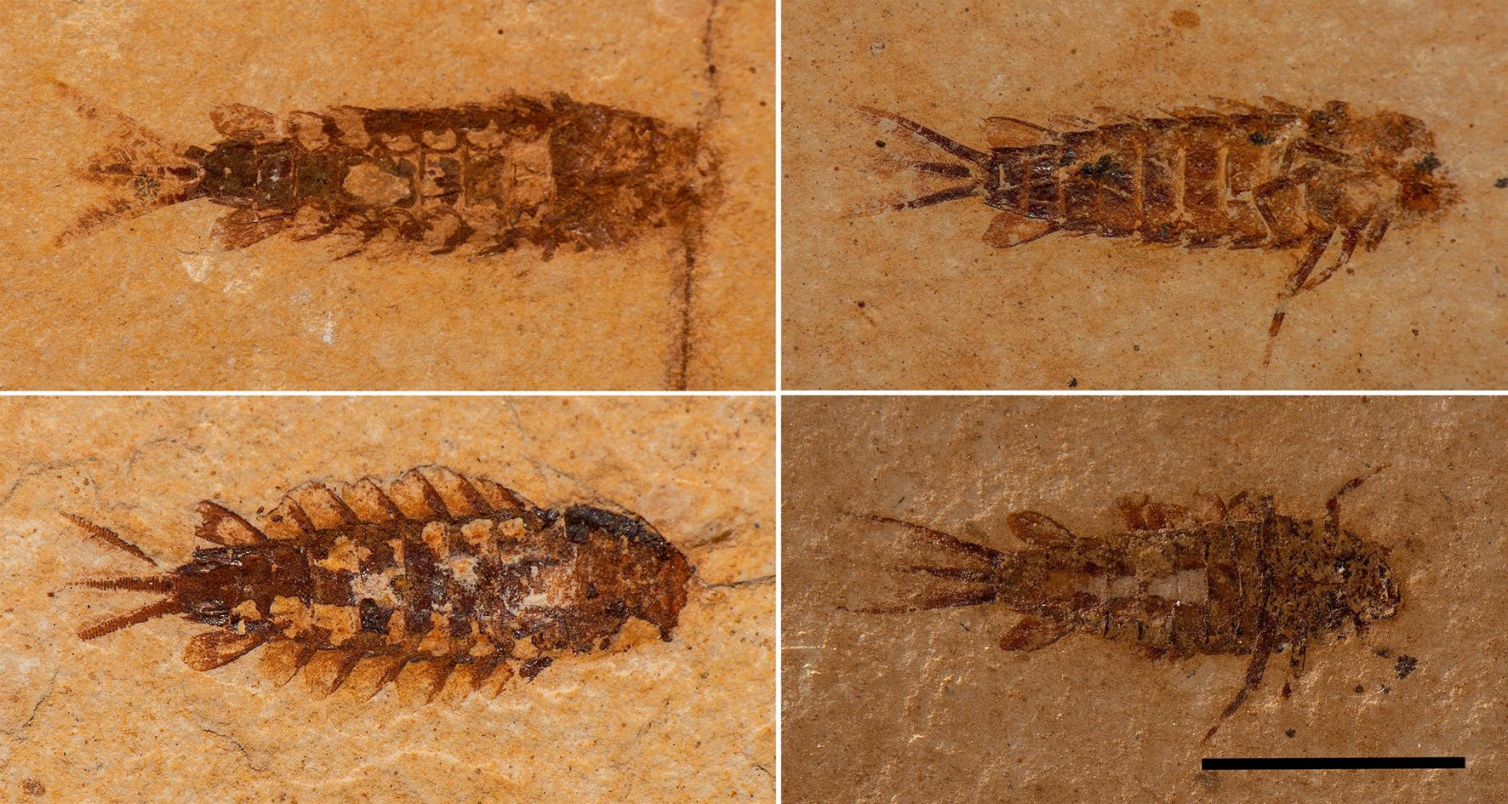
Preservation of larvae from layer 285 cm. Larvae of *Protoligoneuria limai* recovered from layer 285 cm, evidencing the excellent preservation of specimens. Scale bar 5 mm.

**Figure 6:**
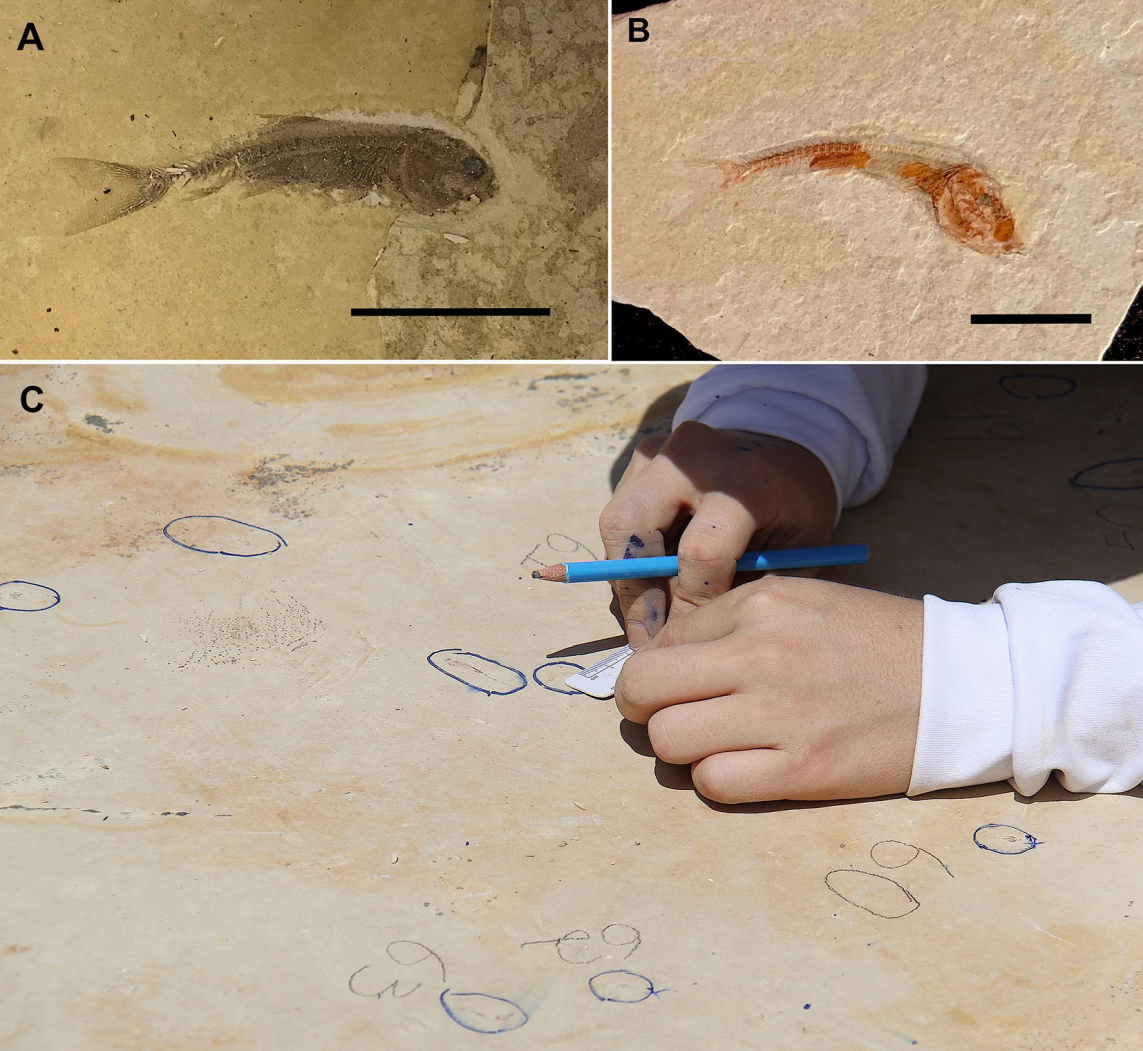
Gonorynchiform fish *Dastilbe*. (**A**) *Dastilbe* specimen recovered at level 205 cm. Scale bar: 25 mm; (**B**) One of the smallest *Dastilbe* specimens recovered at level 285 cm. Scale bar: 5 mm; (**C**) A layer with several *Dastilbe* specimens (inside the blue circles) with preferential orientation. The values written next to the fossils refer to the azimuth.

These larvae have excellent preservation with all specimens complete (head, thorax, abdomen, gills, and cerci preserved—Fig. 5). Also, there is no preferential orientation in the samples suggesting a lack of, or little, transport. The *Dastilbe* individuals from the same level are also smaller than those found in other levels: while they can reach up to 21 cm in length^10^, at the layer 285 cm, the largest one measures 5 cm (Fig. 6), with most of them measuring only 1.5 cm. They are also complete and without preferential orientation.

Individuals belonging to allochthonous taxa that were recovered at the 285 cm layer include plants (complete *Brachyphyllum obesum* Heer, 1881 leaves, one *Araucaria* sp. seed, an incomplete gymnosperm leaf, one *Duartenia araripensis* Mohr et al., 2012 trunk*,* and one incomplete *Pseudofrenelopsis* sp. branch) and terrestrial insects (an incomplete Orthoptera individual, a complete Hemiptera individual, and a complete Blattaria wing) (Fig. 2), all of them also without preferential orientation.

Most of the layers in which Ephemeroptera larvae were found during the controlled excavations presented few individuals, such as one or two. Eighteen larvae were recovered from a layer 180.4 cm from the top of the Formation. However, most individuals had preferential orientation (Supplementary Fig. S1), so this aggregation was probably caused by transport. Also, the number of preserved specimens at this layer was much smaller than that of layer 285 cm.

### Biological community at the controlled excavation

The fossil assemblage of the excavated profile exhibits several groups: plants such as angiosperms (*Iara* sp. and *Choffatia* sp.), gymnosperms (*Araucaria* sp.; *Brachyphyllum* sp.; *Brachyphyllum obesum*; *Duartenia* sp.; *Duartenia araripensis*; *Frenelopsis* sp.; *Ginkgo* sp.; *Lindleycladus* sp.; *Podozamites* sp.; *Pseudofrenelopsis* sp.; *Welwitschia* sp.), and pteridophytes (*Ruffordia goeppertii* Mohr et al., 2007), as well as indeterminate plant logs, charcoal and fungi. Among the fauna, the following groups were recovered: insects (Blattodea/Blattaria and Isoptera; Diptera; Ephemeroptera; Hemiptera; Hymenoptera; Orthoptera), fishes (*Dastilbe* sp., and *Cladocyclus* sp., as well as several unidentified specimens), unidentified shrimps, ichnofossils and feathers.

The most common taxon recovered was the fish *Dastilbe*, representing 79% of all specimens collected during the excavation. The Hexagenitidae larvae came second, with 5% (no adult hexagenitid was recovered). The remaining 16% were plants, coprolites, ichnofossils, and other arthropods and fishes. Mayflies' larvae constituted 85% of the total number of insect specimens excavated.

Almost all recovered *Dastilbe* specimens were juveniles. Fish from other taxa and *Dastilbe* in other ontogenetic stages are present, but are fewer, mainly disarticulated or represented by isolated parts (such as operculum and scales).

In this controlled excavation, there is low species richness in some layers, while in other layers, interchangeably, there is a higher species richness in the assemblage. The richness peaks mostly occur at the frequent *Dastilbe* mass mortality layers (Fig. 2).

## Discussion

Although the local abundance of fossils of a single taxon on a single bedding plane is suggestive of mass mortality^24^, such a conclusion can be reached only when time-averaging processes are discarded as influencers^12^. If on the same bedding plane the taphonomical signatures differ among fossils, as reported to the Green River Formation^25^, then time-averaging is a possible factor for the accumulation. As the analysed taphonomic signatures of all autochthonous individuals of the layer 285 cm are similar and their remains are articulated, we suggest that the mayflies' individuals died simultaneously, representing a mass mortality event.

It is crucial to perform excavations with stratigraphic control to determine whether mass mortality events are extraordinary taphonomic modes within a unit or whether they are more common. Our controlled excavations (Fig. 2) show that, in the Crato Formation, mayfly mass mortality events were rare, though the abundant *Dastilbe* fishes are frequently found in accumulations suggestive of mass mortalities.

There is compelling morphological and taphonomic evidence that hexagenitid larvae were well adapted to standing waters. They have a minnow-like body with posterolateral abdominal processes, lamellar gills with a thickened outer margin and a thickened rib near the posterior margin, slender, weak legs, short claws, and strongly pubescent swimming caudal filaments^26,27^, besides heads that are spherical or oval in dorsal view, and hypognathous in lateral view^28^. Overall, they are very similar to the general appearance of the extant family Siphlonuridae^26^, which inhabits all kinds of aquatic habitats, like lakes, ponds, rivers, swamps, and streams' vegetations^29^. In siphlonurids, minnow-like swimming caudal filaments, as occur in hexagenitids, are associated with quiet-water habitats, and short claws are associated with either quiet waters or habitats with solid rather than fine substrates^26^. Meshkova^27^ concluded that the presence of leaf-shaped gills, weak legs (not adapted for burrowing), and strongly pubescent caudal filaments of the larvae of the hexagenitid *Ephemeropsis* indicated that they had inhabited standing waters. In fact, all hexagenitids from Laurasia have been considered lacustrine^11^. Martins-Neto^9^ described the habitat of several species of Hexagenitidae as consisting of silty and sandy bottoms with running and shallow water or stagnant shallow water within vegetated lakes. Tshernova^30^ and McCafferty^26^ hypothesized quiet waters as a habitat for *Protoligoneuria limai* because of its larval swimming adaptations. It is, therefore, likely that the Crato Formation hexagenitids occurred in quiet waters.

The Hexagenitidae and *Dastilbe* individuals found at layer 285 cm are characterized by excellent preservation with relatively intact specimens. Any transport would have consequences regarding the completeness of morphological elements^8^. Moreover, there is no preferential orientation in the samples; therefore, any transport involving currents or waves is discarded. Braz^31^, studying impressions of the Crato Formation angiosperms, observed that most of the fossils had little fragmentation and concluded that the deposition occurred in a shallow lake environment with little or no transport. Without the action of water transport, the large accumulation verified by us was probably not random but episodic, and such quality of preservation demands a minimal transport distance^32^, agreeing with the hypothesis of an autochthonous fauna.

Exceptional preservation often requires a fast burial caused by an abrupt catastrophic event, in addition to a reduction in oxygenation^22^. Carcasses must also stay far from predators and scavengers to avoid their removal from within the sediment^33^. Moreover, microbially induced sedimentary structures (MISS) could also be important for the preservation of soft tissues^34^. At layer 285 cm, it is possible that the burial of specimens was not due to high sedimentation rates^35^, considering the small size of the *Dastilbe* fishes and Hexagenitidae larvae, which would require a minor sediment cover. In this case, a rapid overgrowth of benthic microbial mats would be enough^34^. Structures similar to MISS were already reported for the Crato Formation; however, they were isolated and without stratigraphic data^5^. Iniesto et al*.*^36^ ran experiments with extant larvae of Coleoptera and microbial mats and, by comparison, showed that grylloids from the Crato Formation had a pattern of preservation consistent with the presence of microbial mats. The latter only occurs in specific situations, such as restricted hypersaline lacustrine settings, shallow water tanks, and in organisms that are rich in lipids, such as insect larvae^9,36,37^.

Based on the fossils found in layer 285 cm, the Hexagenitidae were the main taxon of autochthonous arthropods that managed to survive longer during times of environmental stresses. These larvae are smaller than those found in other levels, suggesting an episode when the water column was so low that they could not moult to reach larval maturity. Younger individuals could support lower water levels due to their small sizes, as in the early stages their body is only 0.1 cm^21^. Furthermore, Kluge^38^ points out that mayflies that develop in warmer waters are smaller than those that live in colder waters. Camp et al*.*^39^ suggested that the climate change and other stressors may make moulting more challenging, since respiratory harms will become more severe at higher temperatures. These authors demonstrated that in the 3–4 hours before moulting, larvae consume 41% more oxygen than normal, and oxygen consumption becomes more extreme at higher temperatures^39^. Thus, given that the larvae spend more oxygen during the moult, this task would be more challenging in a shallow water column, where the oxygen rates are already low^13^. Similarly, we can rule out a post-moult accumulation of mayflies’ exoskeletons, because in the 285 cm layer all larvae are young individuals. It is unlikely that they were mature enough to moult, considering their small sizes and a lack of wing pads^38^. The smaller sizes of the *Dastilbe* individuals found in layer 285 cm are consistent with a shallower water column episode.

**Figure 7:**
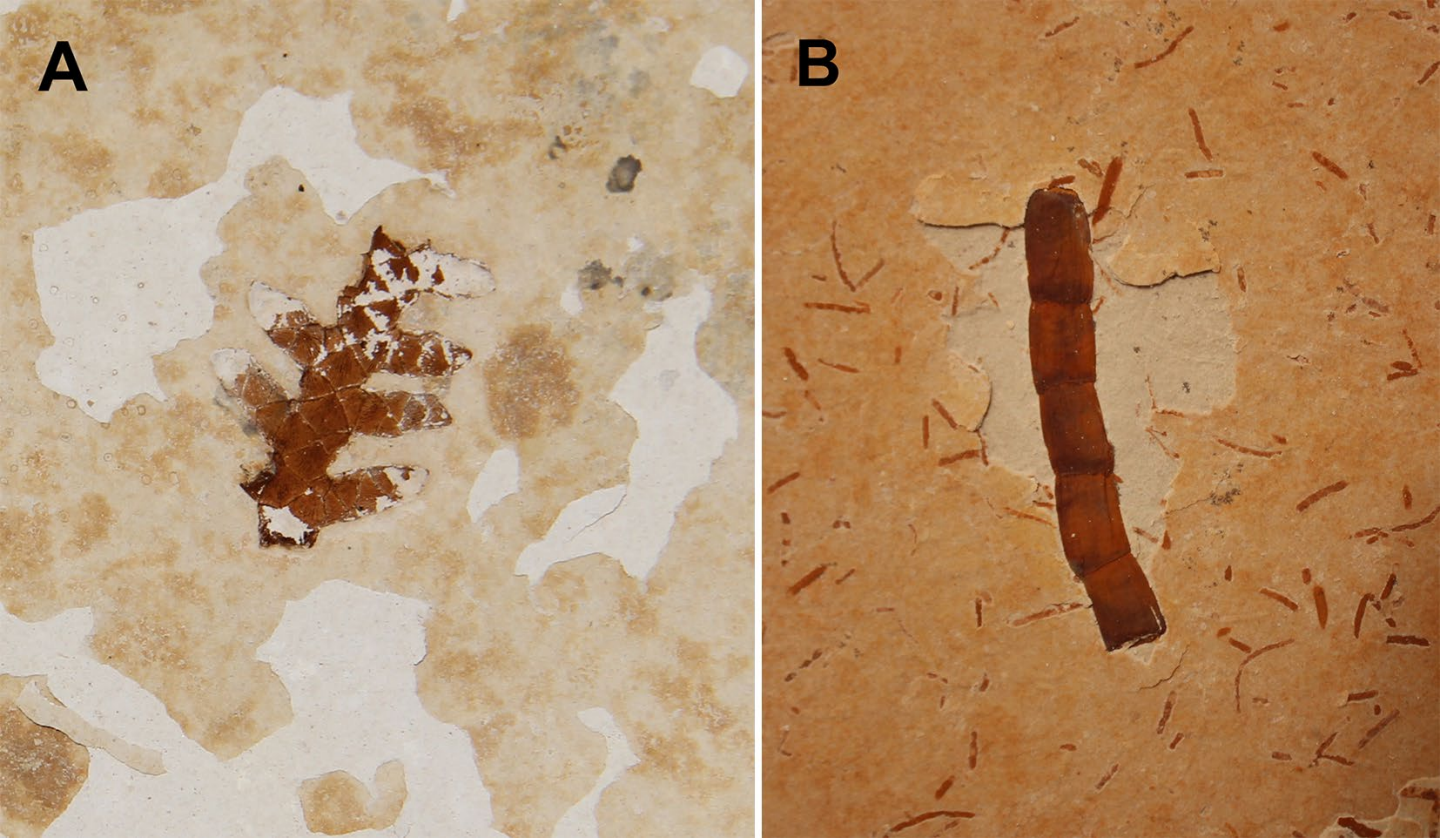
*Brachyphyllum* sp. and *Pseudofrenelopsis* sp. (**A**) *Brachyphyllum* sp. recovered from the controlled excavation; (**B**) *Pseudofrenelopsis* sp. recovered from the controlled excavation.

Gymnosperms constitute the dominant and most diverse group of plants in the Crato palaeoflora, especially the Coniferales^40^. At layer 285 cm, the Coniferales possess xerophytic characters, such as reduced and compressed leaves in *Brachyphyllum obesum* and *Pseudofrenelopsis*^41^ (Fig. 7), as well as thick cuticles, papillae, stomata immersed in the epidermis, and the twisted cauline growth in *Duartenia araripensis*^42^. Their preferred habitat would be coastal, riparian, or marshy sandy regions of saline or brackish water bodies^43^. The presence of *Araucaria* is also related to drier weather conditions^44^. These adaptations to a semi-arid to arid climate support a scenario of significant evaporation at the Crato Formation^45^, a condition under which these plants and the dominant faunas of the palaeolake probably dwelled cyclically^46^.

Over and underlying layers of the mortality level presented crystals and pseudomorphs of halite (NaCl) and lacked fossils of mayflies. Halite forms due to the dissolution of a primary salt precipitate^47^ and its presence could indicate that, with the decrease of the water volume, the salinity of the lake increased and salts precipitated^48^. Macro-invertebrates are considered sensitive indicators of water quality^49^, and their use for that end has long been recognized as effective^50^. Many studies have shown a wide variation in the salinity tolerances of different macro-invertebrate taxa^51–54^. Extant species of mayflies are generally halophobic, and only a few species are reported to tolerate elevated salt concentrations as present in brackish water^55^. Even small increases in salinity will result in the loss of sensitive species^56^ and can lead to salt-tolerant biota gain^57^. Although many taxa may survive at elevated salt concentrations, chronic exposure to increased salinity may significantly reduce juveniles’ recruitment and growth, and the taxa’s reproductive capability^57,58^. The increase of salinity could be a causative agent for the mass mortality of the larvae recovered at level 285 cm, though not necessarily for the *Dastilbe* individuals. Unlike the mayflies’ larvae, modern gonorynchiform fishes (e.g., the 'milkfish' *Chanos chanos* (Forsskål, 1775)) are anadromous and can tolerate varying salinities^59,60^.

Other hypotheses for the death of these mayflies’ larvae are anoxia, temperature and salinization shifts, desiccation, or a combination of factors. Natural modern-day mass mortalities are regular in restricted basins and occur seasonally during dry periods when the surface water temperatures rise, causing salinity oscillation^48^. Sudden turbidity caused by earthquakes or storms cannot be ruled out as causative of layer 285 cm’s mass mortality. However, although seismic events have been proposed for parts of the Araripe Basin due to the presence of wet sediment deformation structures^5^, we have not observed these in the analyzed horizon. Similarly, storm events were reportedly frequent in the Crato Formation due to the presence of storm-damaged plant fragments^47^. Nevertheless, no sedimentological structures compatible with storm events, such tempestites, were seen on the mass mortality layer.

There are a few Lagerstätten whose mayfly fauna can be compared with the Crato Formation, such as the Green River Formation from the Early to Middle Eocene of north-western Colorado and south-western Wyoming, USA^25^; the Koonwarra fossil beds of the Wonthaggi Formation from the Lower Cretaceous of Australia^61^; the Solnhofen beds from the Late Jurassic of Germany^62^; and the Yixian Formation of the lowermost Jehol Group from the Lower Cretaceous of China^63^. The Green River Formation was formed under a temperate to sub-tropical lacustrine setting^64^. The absence of benthic organisms is implied by the lack of bioturbation on the sediment^25^, but there is evidence of microbial mats and a limited nekton^64^. Unfortunately, only its fish have been analyzed taphonomically^64^. The exceptional preservation of the Green River fish fossils was associated in the past with rapid burial^65^, but recent studies state that the carcasses were progressively buried, due to the ‘half and half’ preservation of fishes (only half of the fish is exceptionally preserved)^64^, thus differing from the Crato Formation. Mass mortality events of fishes are also recorded in the Green River Formation, but are limited to few laminae^25^ suggesting that these events were fortuitous and not cyclical as in the Crato paleolake.

The Koonwarra fossil beds represent a freshwater lacustrine or fluvial environment^61^ that had an abundant and diverse insect fauna^61,66^. Although Hemiptera and Coleoptera are the most diverse orders at the unit, the fauna is dominated by aquatic larvae of Ephemeroptera and Diptera^61^ belonging to taxa typical of temperate environments, unlike the tropical Hexagenitidae of the Crato Formation. The preservation of the Koonwarra insects has been extensively discussed, and at first, it was believed that they were preserved in a shallow lake during cold periods, in which the shallower lake portions were isolated by ice and became anoxic^61,67^. Nowadays, it is accepted that, actually, there was a deep lake with a stratified water column^66,67^. Both deposits represent a deep stratified lake, however, there is no evidence of a marine influence or microbial mat in the Koonwarra fossil beds^23,68^. Mass mortality of fishes occurred periodically in the Koonwarra beds^69^, but the events that affected the fish community may not have disturbed the invertebrates^61^, given that no mass mortality has been reported yet for any invertebrate group. The bottom dwelling larvae of *Australurus plexus* Jell and Duncan, 1986 (Ephemeroptera: Siphlonuridae) were considered a common species part of the autochthonous fauna of the Koonwarra beds^61^, as *Protoligoneuria limai* in the Crato Formation. However, unlike the hexagenitids, Siphlonuridae is typical of cool mountain streams and lakes^70^, so the habitat of the Koonwarra depositional site might have been different of that of Crato.

The Solnhofen limestones represent a marine or semi-marine past environment^71^, unlike the Crato Formation that was formed under a lacustrine environment^72^. However, both units have similar modes of mineralization and preservation of fossils^73^. The insects of the Solnhofen beds have been extensively examined, but mainly restricted to taxonomic studies. The Solnhofen entomofauna presents adult Odonata as their most numerous insects, but their larvae have not been found, likely due to the hypersaline paleoenvironment^62^. Mayflies are numerous as well, but only as adults^62^. However, because these adults usually are not able to fly long distances^74^, they were probably buried close to the original freshwater habitat of their larvae.

One of the most species-rich Mesozoic Lagerstätte is the Yixian Formation^75^. Its deposits are known for the exceptional preservation of fossils, due to periodic anoxia, volcanic input, and rapid burial^76^. As seen in the Crato Formation, cyclical mass mortality events of fishes are present^77^. In the Yixian Formation, the main cause was periodic anoxia in the coldest seasons, with re-oxygenation in warmer seasons^77^. Pan et al*.*^78^, analyzing the *Ephemeropsis trisetalis* Eichwald, 1864 (Hexagenitidae) collected under stratigraphically controlled excavations, found that this group is one of the most abundant in the Jehol Biota (the vast majority as larvae). *E. trisetalis* occurs in various preservational states, but most specimens are fully articulated^78^. Biostratinomic and paleoecological studies of *E. trisetalis* larvae indicate that they were autochthonous and preserved under low energy conditions^77,78^, like *P. limai* in the Crato Formation.

In our controlled excavation, *Dastilbe* were often found in mass mortality events, agreeing with a scenario in which such mortality events were cyclical^12^. The Crato Formation probably acted as a nursery for this species, with the adults migrating to reproduce^60^, as virtually all specimens recovered were juvenile. It is possible that these juveniles were the only fish continually at the paleolake at the analysed area^60^, since fish from other taxa and *Dastilbe* in other ontogenetic stages are rarer, and mainly disarticulated or representing isolated parts, and could represent carcasses that were transported into the excavated locality.

Previously, mayflies were pointed out by Menon and Martill^8^ as constituting around 14%–24% (adults and larvae) of the total insect diversity of the Crato Formation, unlike Bechly^72^ that previously have reported only 7%. We found in this controlled excavation that mayflies' larvae constituted 85% of the total number of insect specimens excavated. These low percentages previously found are probably due to taxonomically biased collections and/or absence of excavations with stratigraphic control.

## Conclusions

According to Martins-Neto^9,13^, at least one group of insects experienced mass mortality episodes in the Crato palaeolake: Hexagenitidae larvae. There is robust evidence to consider the assemblage at layer 285 cm as such. The palaeoenvironment of the Crato palaeolake was subject to constant shifts in salinity, water depth, and degree of oxygenation, and this likely seasonal phenomenon of high evaporation^46^, probably caused by the hot climate tending to aridity, could have caused stress on the aquatic animals, as already pointed out by several authors^47,79–84^. Such environmental scenario possibly resulted in this punctual mass mortality. Notwithstanding, a more detailed analysis of environmental proxies is urgent to interpret biological crises in the Araripe Basin better. Excavations with stratigraphic control at the Crato Formation provide essential data to understand major tendencies in its ancient biological community. The dominant taxon found in the controlled excavation was the gonorynchiform fish *Dastilbe,* followed by the Hexagenitidae larvae, representing the best candidates for quantitative studies in the Crato Formation. As mayfly fossils represent part of the lake’s autochthonous fauna, data collected from them, along with palaeoclimatic, sedimentological, and biological observations, can be used to understand the palaeoenvironmental context of this unit better.

## Supplementary Information

Supplementary Figure S1.

Supplementary Table S1.

## Data Availability

All data generated or analysed during this study are included in this published article (and its “Supplementary Information” files).
