# Supplementary material for: Mass mortality events of autochthonous faunas in a Lower Cretaceous Gondwanan Lagerstätte
Source: Sci Rep. 2021 Mar 26;11:6976. doi: 10.1038/s41598-021-85953-5 (PMC7997927; doi:10.1038/s41598-021-85953-5)
Supplement: Supplementary file 1 — Supplementary Figure S1. [file 41598_2021_85953_MOESM1_ESM.docx]

**Mass mortality events of autochthonous faunas in a Lower Cretaceous Gondwanan Lagerstätte**

ARIANNY P STORARI^1*^, TAISSA RODRIGUES^1^, RENAN AM BANTIM^2^, FLAVIANA J LIMA^2^ & ANTONIO AF SARAIVA^2^

¹Laboratório de Paleontologia, Departamento de Ciências Biológicas, Centro de Ciências Humanas e Naturais, Universidade Federal do Espírito Santo, Vitória, Espírito Santo, Brazil; ORCID IDs: 0000-0002-3645-3015 (APS), 0000-0001-7918-1358 (TR); e-mails: ariannystorari@gmail.com*, taissa.rodrigues@ufes.br

^2^Laboratório de Paleontologia da URCA - LPU, Centro de Ciências Biológicas e da Saúde, Universidade Regional do Cariri, Crato, Ceará, Brazil; ORCID IDs: 0000-0003-4576-0989 (RAMB), 0000-0001-8602-6508 (FJL), 0000-0003-0127-8912 (AAFS); e-mails: renanbantimbiologo@gmail.com, flavianajorge@gmail.com, alamocariri@gmail.com


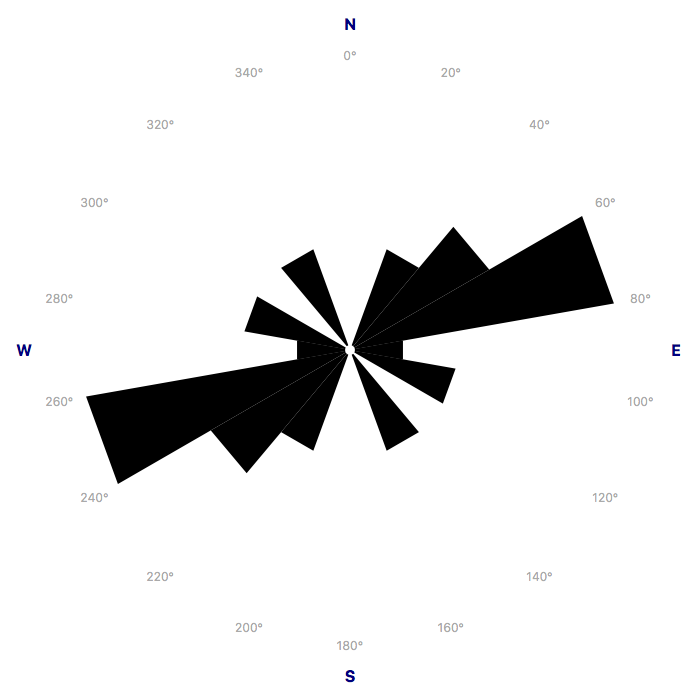


**Supplementary Figure S1. Layer 180.4 rose diagram.** Rose diagram produced with GeoRose 0.5.1.1 software, showing the mayfly's fossil directions present on layer 180.4.
