# Supplementary material for: Mass mortality events of autochthonous faunas in a Lower Cretaceous Gondwanan Lagerstätte
Source: Sci Rep. 2021 Mar 26;11:6976. doi: 10.1038/s41598-021-85953-5 (PMC7997927; doi:10.1038/s41598-021-85953-5)
Supplement: Supplementary file 2 — Supplementary Table S1. [file 41598_2021_85953_MOESM2_ESM.docx]

**Mass mortality events of autochthonous faunas in a Lower Cretaceous Gondwanan Lagerstätte**

ARIANNY P STORARI^1*^, TAISSA RODRIGUES^1^, RENAN AM BANTIM^2^, FLAVIANA J LIMA^2^ & ANTONIO AF SARAIVA^2^

¹Laboratório de Paleontologia, Departamento de Ciências Biológicas, Centro de Ciências Humanas e Naturais, Universidade Federal do Espírito Santo, Vitória, Espírito Santo, Brazil; ORCID IDs: 0000-0002-3645-3015 (APS), 0000-0001-7918-1358 (TR); e-mails: ariannystorari@gmail.com*, taissa.rodrigues@ufes.br

^2^Laboratório de Paleontologia da URCA - LPU, Centro de Ciências Biológicas e da Saúde, Universidade Regional do Cariri, Crato, Ceará, Brazil; ORCID IDs: 0000-0003-4576-0989 (RAMB), 0000-0001-8602-6508 (FJL), 0000-0003-0127-8912 (AAFS); e-mails: renanbantimbiologo@gmail.com, flavianajorge@gmail.com, alamocariri@gmail.com

**Supplementary Table S1. Lithostratigraphic position of the mayflies’ larvae assemblage.** The table indicates the quadrant of collection (distance from the top of the excavation in cm); integrity of the specimen (complete, incomplete or fragment); type of preservation; length in cm; orientation (azimuth). The 'x' in the azimuth column is for no orientation.

| **Quadrant** | **Integrity** | **Preservation** | **Length** | **Azimuth** |
| --- | --- | --- | --- | --- |
| 11.2 | Incomplete | Impression | 0.3 | x |
| 19.5 | Incomplete | Impression | 0.5 | x |
| 95.8 | Complete | Compressed | 0.8 | x |
| 130.4 | Incomplete | Compressed | 0.5 | x |
|  | Complete | Compressed | 0.7 | 330 |
|  | Complete | Compressed | 1.0 | x |
| 138 | Complete | Compressed | 1.0 | 270 |
| 151.4 | Incomplete | Compressed | 0.5 | x |
| 156 | Complete | Compressed | 0.8 | x |
| 180.4 | Complete | Compressed | 1.0 | 73 |
|  | Complete | Compressed | 1.2 | 44 |
|  | Complete | Compressed | 1.3 | 260 |
|  | Complete | Substitution | 0.8 | 66 |
|  | Complete | Substitution | 1.3 | 205 |
|  | Complete | Substitution | 0.9 | 334 |
|  | Complete | Substitution | 1.5 | 290 |
|  | Incomplete | Substitution | 0.7 | 76 |
|  | Complete | Substitution | 1.0 | 232 |
|  | Complete | Substitution | 0.8 | x |
|  | Complete | Substitution | 0.8 | 66 |
|  | Complete | Substitution | 1.3 | 205 |
|  | Complete | Substitution | 0.9 | 334 |
|  | Complete | Substitution | 1.5 | 290 |
|  | Incomplete | Substitution | 0.7 | 76 |
|  | Complete | Substitution | 1.0 | 232 |
|  | Complete | Substitution | 0.8 | x |
|  | Complete | Substitution | 0.8 | 97 |
| 180.5 | Complete | Substitution | 1.1 | x |
|  | Complete | Substitution | 1.2 | x |
|  | Complete | Substitution | 1.1 | 150 |
|  | Complete | Substitution | 1.2 | x |
|  | Complete | Substitution | 1.2 | x |
|  | Complete | Substitution | 1.4 | x |
|  | Complete | Substitution | 1.1 | x |
|  | Complete | Substitution | 1.1 | x |
|  | Complete | Substitution | 1.2 | x |
|  | Complete | Substitution | 1.3 | x |
|  | Complete | Substitution | 1.1 | x |
|  | Complete | Substitution | 1.2 | x |
|  | Complete | Substitution | 1.1 | 150 |
| 180.9 | Complete | Substitution | 1.1 | x |
| 181 | Complete | Compressed | 0.8 | x |
|  | Complete | Compressed | 1.0 | x |
|  | Complete | Compressed | 1.0 | x |
|  | Complete | Compressed | 1.0 | x |
|  | Complete | Compressed | 1.0 | x |
|  | Complete | Compressed | 1.0 | x |
|  | Incomplete | Compressed | 0.8 | x |
|  | Complete | Compressed | 1.0 | x |
|  | Complete | Compressed | 1.0 | x |
| 183 | Complete | Substitution | 0.7 | x |
|  | Complete | Substitution | 1,3 | x |
| 185.8 | Complete | 3D | 1.5 | x |
| 186.3 | Incomplete | 3D | 1.0 | x |
|  | Complete | Compressed | 1.5 | x |
|  | Complete | Impression | 1.0 | x |
|  | Complete | Compressed | 1.0 | x |
|  | Complete | Compressed | 1.5 | x |
|  | Complete | 3D | 1.5 | x |
|  | Complete | 3D | 1.8 | x |
| 187.1 | Complete | Compressed | 1.0 | x |
|  | Incomplete | Compressed | 1.0 | x |
| 187.3 | Incomplete | Compressed | 0.9 | x |
|  | Complete | Compressed | 1.7 | x |
| 189.3 | Complete | Compressed | 0.8 | x |
|  | Complete | Compressed | 0.9 | x |
|  | Complete | Impression | 0.8 | x |
|  | Complete | Impression | 1.0 | x |
|  | Incomplete | Compressed | 1.0 | x |
|  | Incomplete | Compressed | 0.5 | x |
|  | Incomplete | Compressed | 0.5 | x |
|  | Complete | Compressed | 0.9 | x |
|  | Complete | Compressed | 1.0 | x |
|  | Complete | Compressed | 0.9 | x |
|  | Complete | Compressed | 1.0 | x |
|  | Complete | Compressed | 1.0 | x |
| 191.3 | Complete | Impression | 1.0 | x |
| 192 | Complete | Compressed | 1.0 | x |
|  | Complete | Compressed | 1.6 | x |
|  | Complete | Compressed | 1.6 | x |
| 192,5 | Complete | Compressed | 1.2 | x |
|  | Complete | Compressed | 0.9 | x |
| 192.9 | Complete | Compressed | 1.1 | x |
|  | Complete | Compressed | 1.2 | x |
| 193 | Complete | Compressed | 1.6 | x |
|  | Complete | Compressed | 1.0 | x |
|  | Complete | Compressed | 1.2 | x |
|  | Complete | Compressed | 1.1 | x |
| 193.4 | Incomplete | Compressed | 0.1 | x |
|  | Incomplete | Compressed | 0.6 | x |
|  | Complete | Compressed | 1.3 | x |
|  | Complete | Compressed | 1.0 | x |
|  | Complete | Compressed | 1.0 | x |
| 195 | Complete | Compressed | 1.0 | x |
|  | Complete | Compressed | 1.0 | x |
|  | Complete | Compressed | 1.5 | x |
|  | Complete | Compressed | 1.0 | x |
| 199 | Complete | Impression | 1.0 | x |
| 201 | Complete | Compressed | 1.2 | x |
| 203.5 | Complete | Compressed | 1.0 | x |
| 205 | Incomplete | Compressed | 1.0 | x |
|  | Complete | Compressed | 1.0 | x |
|  | Complete | Compressed | 1.0 | x |
| 220 | Complete | Compressed | 1.7 | x |
|  | Complete | Compressed | 1.7 | x |
| 224 | Complete | Compressed | 1.0 | x |
|  | Incomplete | Compressed | 0.5 | x |
|  | Complete | Compressed | 1.0 | x |
| 265 | Complete | Compressed | 1.0 | x |
|  | Complete | Compressed | 1.0 | x |
| 285 | Complete | Compressed | 0.8 | x |
|  | Complete | Compressed | 0.8 | x |
|  | Complete | Compressed | 0.8 | x |
|  | Complete | Compressed | 0.8 | x |
|  | Complete | Compressed | 0.8 | x |
|  | Complete | Compressed | 0.8 | x |
|  | Complete | Compressed | 1.0 | x |
|  | Complete | Compressed | 1.0 | x |
|  | Complete | Compressed | 0.6 | x |
|  | Complete | Compressed | 0.6 | x |
|  | Complete | Compressed | 1.0 | x |
|  | Complete | Compressed | 1.0 | x |
|  | Complete | Compressed | 0.8 | x |
|  | Complete | Compressed | 1.0 | x |
|  | Complete | Compressed | 1.0 | x |
|  | Complete | Compressed | 1.0 | x |
|  | Complete | Compressed | 1.0 | x |
|  | Complete | Compressed | 1.0 | x |
|  | Complete | Compressed | 1.0 | x |
|  | Complete | Compressed | 1.0 | x |
|  | Complete | Compressed | 1.0 | x |
|  | Complete | Compressed | 1.0 | x |
|  | Complete | Compressed | 1.0 | x |
|  | Complete | Compressed | 1.0 | x |
|  | Complete | Compressed | 1.0 | x |
|  | Complete | Compressed | 0.4 | x |
|  | Complete | Compressed | 0.4 | x |
|  | Complete | Compressed | 0.5 | x |
|  | Complete | Compressed | 0.5 | x |
|  | Complete | Compressed | 0.5 | x |
|  | Complete | Compressed | 0.5 | x |
|  | Complete | Compressed | 1.0 | x |
|  | Complete | Compressed | 1.0 | x |
|  | Complete | Compressed | 1.0 | x |
|  | Complete | Compressed | 1.0 | x |
|  | Complete | Compressed | 1.0 | x |
|  | Complete | Compressed | 1.0 | x |
|  | Complete | Compressed | 1.0 | x |
|  | Complete | Compressed | 1.0 | x |
| 309 | Complete | Compressed | 1.5 | x |
